# Comparative biogeography of the gut microbiome between Jinhua and Landrace pigs

**DOI:** 10.1038/s41598-018-24289-z

**Published:** 2018-04-13

**Authors:** Yingping Xiao, Fanli Kong, Yun Xiang, Weidong Zhou, Junjun Wang, Hua Yang, Guolong Zhang, Jiangchao Zhao

**Affiliations:** 1 Institute of Quality and Standards for Agro-products, Zhejiang Academy of Agricultural Sciences, Hangzhou, Zhejiang, 310021 China; 2 Department of Animal Science, University of Arkansas, Fayetteville, Arkansas 72701 USA; 3 Isotope Research laboratory, Colloge of Life Science, Sichuan Agricultral University, Ya’an, Sichuan 625014 China; 4 Institute of Animal Husbandry and Veterinary Medicine, Jinhua Academy of Agricultural Sciences, Jinhua, Zhejiang, 321017 China; 5 Institute of Animal Husbandry and Veterinary Medicine, Zhejiang Academy of Agricultural Sciences, Hangzhou, Zhejiang, 321017 China; 6 Beijing Advanced Innovation Center for Food Nutrition and Human Health, State Key Laboratory of Animal Nutrition, China Agricultural University, Beijing, 100094 China; 7 Department of Animal Science, Oklahoma State University, Stillwater, Oklahoma 74078 USA

## Abstract

The intestinal microbiome is critically important in shaping a variety of host physiological responses. However, it remains elusive on how gut microbiota impacts overall growth and more specifically, adipogenesis. Using the pig as an animal model, we compared the differences in bacterial community structure throughout the intestinal tract in two breeds (Landrace and Jinhua) of pigs with distinct phenotypes. The Landrace is a commercial purebred and the Jinhua is a Chinese indigenous, slow-growing breed with high propensity for fat deposition. Using 16S rRNA gene sequencing, we revealed that the bacterial communities are more diverse in the duodenum, jejunum, and cecum of Jinhua pigs than in those of Landrace pigs, whereas the ileal and colonic microbiota show a similar complexity between the two breeds. Furthermore, a number of bacterial taxa differentially exist in Jinhua and Landrace pigs throughout the entire intestinal tract, with the jejunal and ileal microbiome showing the greatest contrast. Functional prediction of the bacterial community suggested increased fatty acid biosynthesis in Jinghua pigs, which could partially explain their adiposity phenotype. Further studies are warranted to experimentally verify the relative contribution of each enriched bacterial species and their effect on adipogenesis and animal growth.

## Introduction

Pigs are an important livestock species that serve as both a major food source and an important animal model to study human diseases due to their large size and similar anatomy to humans^1^. Several recent studies characterized the gut microbiome in pigs regarding its relationship with diet^2–4^, developmental stage^5^, lipid metabolism^6^, antibiotic resistance^7^, meat quality^8^, feed efficiency^9^, and growth performance^10^. However, relatively little is known about the biogeography of gut microbiota in pigs and their correlation with adipogenesis.

The objective of this study was to characterize and compare the biogeography of gut microbiota in two swine breeds with distinct phenotypes, namely Jinhua and Landrace pigs. The Jinhua pig is a well-known indigenous breed of Zhejiang Province in eastern China with a slow growth rate but a high propensity for adipogenesis and intramuscular fat deposition^11,12^. In contrast, the Landrace pig is a lean, fast-growing breed of Danish origin selected for high carcass yield^13^. To understand the correlation between intestinal microbiota, swine growth and adipogenesity, we compared the biogeography of gut microbiotas collected from different segments of the intestinal tract of Jinhua and Danish Landrace pigs.

## Materials and Methods

### Animals and sample collection

All animal procedures were approved by the Institutional Animal Care and Use Committee of the Zhejiang Academy of Agricultural Sciences and all methods were performed in accordance with the relevant guidelines and regulations. Three Jinhua and three Landrace sows of a similar age were selected, fed the same diet, and housed in an environmentally controlled room. After parturition, a total of 36 weanling pigs, with three males and three females of similar weight from each sow, were selected and weaned on day 35. All piglets were then housed in six pens in an environmentally controlled room and fed ad libitum commercial diet under standard management, with six pigs from each sow housed in a single pen. Five healthy male and five female piglets of similar weight were selected from each breed and slaughtered on day 240. The digesta from the middle of the duodenum, jejunum, cecum, ileum and colon were collected, snap frozen in liquid nitrogen, and stored at −80 °C. The average thickness of the backfat was measured on the first rib, last rib, and last lumbar vertebrae in the midline using a sliding caliper as described^14^. Intramuscular fat in the longissimus muscle was also measured according to the AOAC (1990) procedures.

### DNA extraction, sequencing, and data analyses

Bacterial DNA was isolated from each intestinal digesta using the QIAamp DNA Stool Mini Kit (QIAGEN, CA) according to the manufacturer’s instructions. The concentration and purity of the extracted DNA was measured using a NanoDrop ND-1000 Spectrophotometer (NanoDrop, Germany), and the integrity was evaluated on a 1% (w/v) agarose gel. The V4 hypervariable region of the 16S rRNA gene was then amplified with degenerate primers, 515 F (5′-GTGCCAGCMGCCGCGGTAA-3′) and 806 R (5′-GGACTACHVGGGTWTCTAAT-3′)^15^. The PCR reaction was carried out using Phusion High-Fidelity PCR Master Mix (New England Biolabs) under the following condition: initial denaturation at 98 °C for 1 min, followed by 30 cycles of denaturation at 98 °C for 10 s, annealing at 50 °C for 30 s, and elongation at 72 °C for 30 s, and a final extension at 72 °C for 5 min. PCR products were purified using GeneJET Gel Extraction Kit (Thermo Scientific). Amplicons from different samples were mixed in equal amounts. Libraries were generated by using the Illumina TruSeq DNA PCR-Free Library preparation kit and sequenced by Novogene (Beijing, China) on Illumina HiSeq 2500.

The datasets generated during the current study are available in the MG-RAST repository (http://metagenomics.anl.gov/mgmain.html?mgpage=project&project=mgp82198).

Sequences were processed and analyzed using mothur v.1.38.0^16^ following MiSeq SOP (http://www.mothur.org/wiki/MiSeq_SOP)^17^. The 2 × 250 bp paired-end reads with ambiguous bases or longer than 275 bp were removed. The UCHIME^18^ and preclustering methods^19^ were used to remove chimeric sequences and to reduce sequencing noise, respectively. Sequences related to Archaea, Chloroplast, Mitochondria, and Eukaryota were removed according to the MiSeq SOP. Singletons were removed to reduce the size of the distance matrix. Sequences were aligned with the SILVA reference database (Full length sequences and taxonomy references release 119, https://www.arb-silva.de/documentation/release-119/)^20^ and assigned to operational taxonomic units (OTUs) based on the sequence identity threshold of 97% and were classified using the RDP classifier^21^.

### Statistical analyses

To reduce the biases caused by the sequencing depth, the reads of each sample were randomly subsampled to 51,612 for downstream α- and β-diversity analyses. The α-diversity was examined using Shannon Index^22^. The Bray-Curtis distance^23^ was calculated to estimate the dissimilarity in the community structure, which was visualized using principal coordinates analysis (PCoA). Analysis of similarities (ANOSIM) was performed in mothur v1.380. Enriched OTUs of gut microbiota in both Jinhua and Landrace pigs were identified using LEfSe (Linear discriminant analysis Effect Size) (http://huttenhower.sph.harvard.edu/galaxy/)^24^, which first compares the relative abundance of OTUs using the Kruskal-Wallis test at an alpha level of 0.05. The resulting significantly different OTUs were then used as input for linear discriminant analysis (LDA) to calculate their effect sizes. OTUs with the logarithmic LDA score threshold of >2.0 were selected as significantly different OTUs between groups. PICRUSt was further utilized to infer the functional pathways from 16 S rRNA gene sequencing data and the Greengenes database 13.5^25^. The statistical differences between pairs of sample or multiple groups of KEGG pathways were visualized using the STAMP (Statistical Analysis of Metagenomic Profiles) Software package^26^.

ANOVA test was used to test the differences in alpha diversity. Significance was set at *p* < 0.05.

## Results

### Growth performance and fat deposition in Jinhua and Landrace pigs

**Figure 1:**
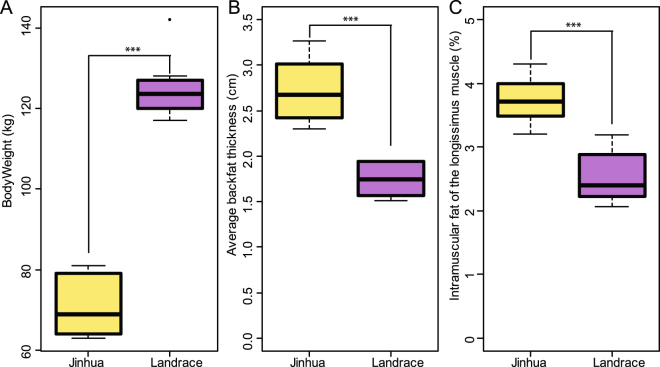
Differences in body weight (**A**), backfat thickness (**B**) and intramuscular fat (**C**) between 240-day-old Jinhua and Landrace pigs. ***P < 0.001 (by unpaired Student’s *t*-test).

Both Jinhua and Landrace piglets were weaned on the same day and fed the same commercial diet under a common standard management. On day 240 the average body weight of Landrace pigs was much higher than that of Jinhua pigs (124.5 kg vs. 70.6 kg; *P* < 0.001; Fig. 1A). However, Jinhua pigs showed a much stronger capacity for fat deposition. The average backfat thickness of Jinhua pigs was much higher than that of Landrace pigs (2.7 cm vs. 1.7 cm; *P* < 0.001; Fig. 1B). Similarly, Jinhua pigs had a higher percentage of the fat in the *Longissimus* muscle than Landrace pigs (3.74% vs. 2.55%; *P* < 0.001; Fig. 1C).

### α-diversity

**Figure 2:**
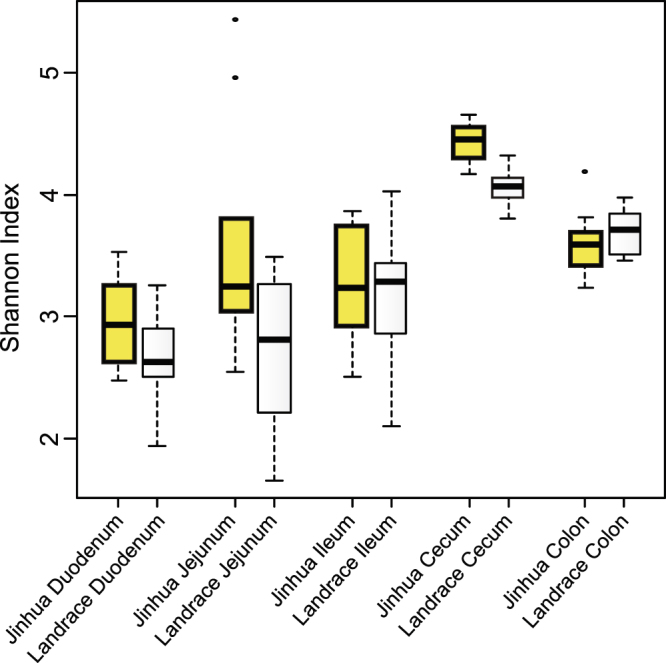
Community diversity in different intestinal segments of Jinhua and Landrace pigs. The cecum harbors the most diverse microbiome among all intestinal segments in both Jinhua and Landrace pigs. Jinhua pigs host more diverse bacterial communities in the duodenum, jejunum, and cecum than Landrace pigs.

To reveal potential differences in the intestinal microbiota composition between Jinhua and Landrace pigs, 16S rRNA gene sequencing was performed using bacterial DNA isolated from the digesta of different intestinal segments of 240-day-old pigs. An obvious difference in community diversity was observed between Jinhua and Landrace pigs. Within each breed, the cecum harbored the most diverse bacterial communities among different intestinal segments as revealed by the Shannon index (Fig. 2). Comparing both breeds, the microbiomes in the duodenum, jejunum, and cecum of Jinhua pigs were more diverse than those of Landrace pigs, whereas the diversity in the ileum and colon was similar (Fig. 2).

### β-diversity: within breed

**Figure 3:**
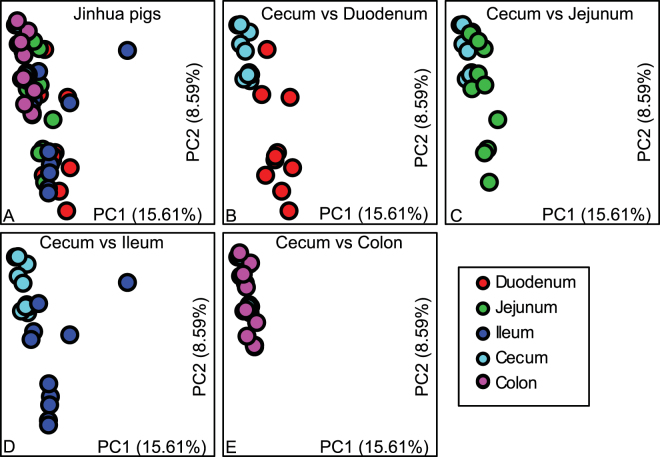
Biogeography of gut microbiome in Jinhua pigs. Principal coordinate analysis (PCoA) shows bacterial community structures based on Bray-Curtis distances. On the PCoA plot, each symbol represents one gut microbiome. (**A**) The microbiota composition in five intestinal segments in Jinhua pigs. (**B**–**E**) Pair-wise comparison between the cecal microbiota vs the microbiota in the duodenum, jejunum, cecum and colon, respectively. The numbers of PC1 and PC2 shows the percent variation explained by the PCoA plot.

**Figure 4:**
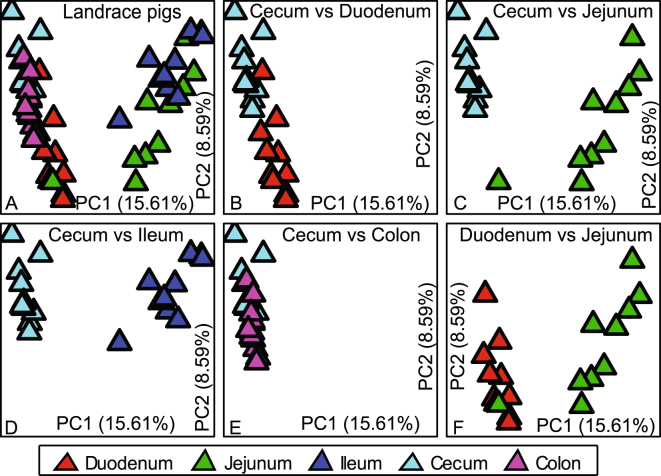
Biogeography of the gut microbiome in Landrace pigs. Principal coordinate analysis (PCoA) shows bacterial community structures based on Bray-Curtis distances. On the PCoA plot, each symbol represents one gut microbiome. (**A**) Microbiota in five intestinal segments in Landrace pigs. (**B**–**E**) show pair-wise comparison between the cecal microbiota vs the microbiota in the duodenum, jejunum, cecum and colon, respectively. (**F**) Microbiota in the duodenum and jejunum of Landrace pigs. The numbers of PC1 and PC2 shows the percent variation explained by the PCoA plot.

Different patterns of the gut microbiome biogeography were observed in Jinhua and Landrace pigs. In Jinhua pigs, the microbiomes of different intestinal segments clustered together except for the cecal microbiome. Albeit with no obvious separation in the bacterial community structure among the duodenum, jejunum, and ileum, a significant difference was seen between the cecum and any of the three small intestinal segments in Jinhua pigs (Fig. 3, Table S1, ANOSIM, P < 0.001). Similarly, the cecal microbiome in Landrace pigs was also distinct from the duodenal, jejunal and ileal microbiomes in Landrace pigs (Fig. 4, Table S1, ANOSIM, P < 0.001). Significant differences in the cecal and colonic microbiomes were observed in both breeds (Table S1). However, the bacterial community structures showed more variation among different segments of the intestinal tract in Landrace than Jinhua pigs (Fig. 4). For example, the microbiomes in the duodenum and jejunum formed distinct clusters in Landrace pigs (Fig. 4).

### β-diversity: between breeds

**Figure 5:**
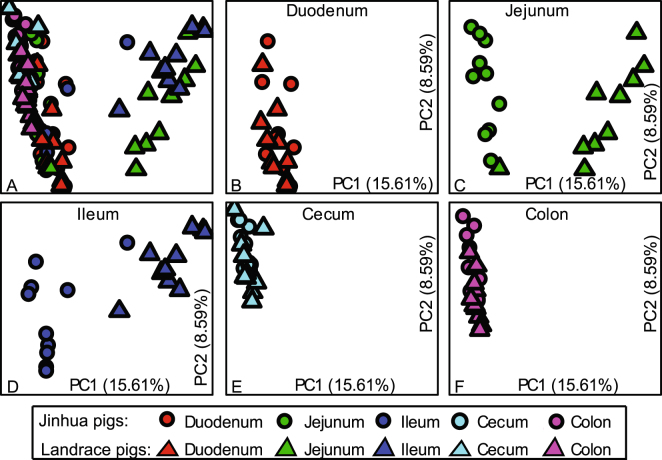
Biogeography of gut microbiome between Jinhua (circles) and Landrace (triangles) pigs. Principal coordinate analysis (PCoA) shows bacterial community structures based on Bray-Curtis distances. On the PCoA plot, each symbol represents one gut microbiome. (**A**) Gut microbiota in five intestinal segments in both Jinhua and Landrace pigs. (**B**–**F**) Pair-wise comparison (Jinhua vs Landrace) of the microbiota in duodenum, jejunum, ileum, cecum and colon, respectively. The numbers of PC1 and PC2 shows the percent variation explained by the PCoA plot.

Cross-sectional comparison of the microbiomes in the same intestinal segment between Jinhua and Landrace pigs revealed that the duodenal, cecal, and colonic microbiomes showed little variation, on the other hand, the jejunal and ileal microbiomes were significantly different between the two breeds (Fig. 5; Table S1).

### Community composition

**Figure 6:**
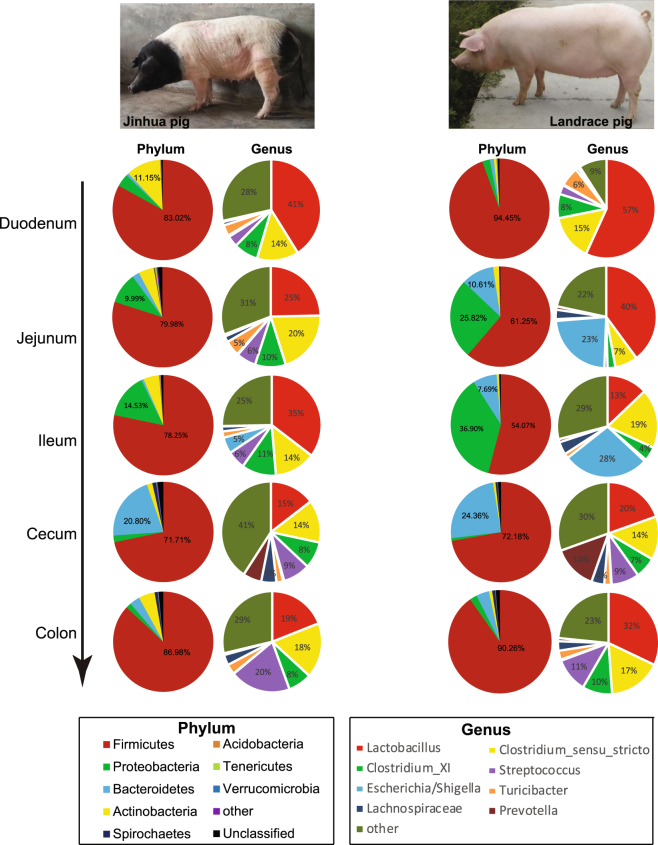
Community composition of the gut microbiota in different intestinal segments of Jinhua and Landrace pigs at the phylum and genus levels, respectively.

At the phylum level, the bacterial taxa varied greatly in different segments of the intestinal tract. Among these taxa, Firmicutes were the most predominant, representing 54.94% of all bacterial populations in both Jinhua and Landrace pigs (Fig. 6 and Figure S1). Proteobacteria constituted second most abundant phylum in the jejunum and ileum ranging from 10 to 37%, whereas Bacteroidetes ranked the second in the cecum (21–24%) in both breeds. All other phyla were relatively minor, representing generally <10% of the bacterial populations throughout the intestinal tract of both breeds. Between the two breeds, Actinobacteria were evidently more abundant in Jinhua than Landrace pigs in each intestinal segment. Furthermore, a high abundance of Bacteroidetes and Proteobacteria were observed in the jejunum and ileum of Landrace compared to those in Jinhua pigs (Fig. 6 and Figure S1).

At the genus level, *Lactobacilli* and *Clostridia* were generally the two most abundant genera in all intestinal segments of both breeds, representing 36–80% of all bacteria when combined (Fig. 6 and Figure S2). However, bacterial taxa were quite different throughout the intestinal tract between Jinhua and Landrace pigs. It was particularly true in the jejunum and ileum, where an *Escherichia* species was much more dominant in Landrace than in Jinhua pigs (23–28% vs. <5%) (Fig. 6). On the other hand, *Streptococci* constituted 6% of all bacteria in the jejunum or ileum of Jinhua pigs, while virtually absent in the same segments of Landrace pigs. It is noteworthy that *Streptococci* were as dominant as *Lactobacilli* or *Clostridia* in the colon of Jinhua pigs, representing approximately 20% of the bacteria present, but made up only 11% of the bacteria in the colon of Landrace pigs (Fig. 6, Figure S2).

### Bacterial taxa differentially represented in Jinhua and Landrace pigs

**Figure 7:**
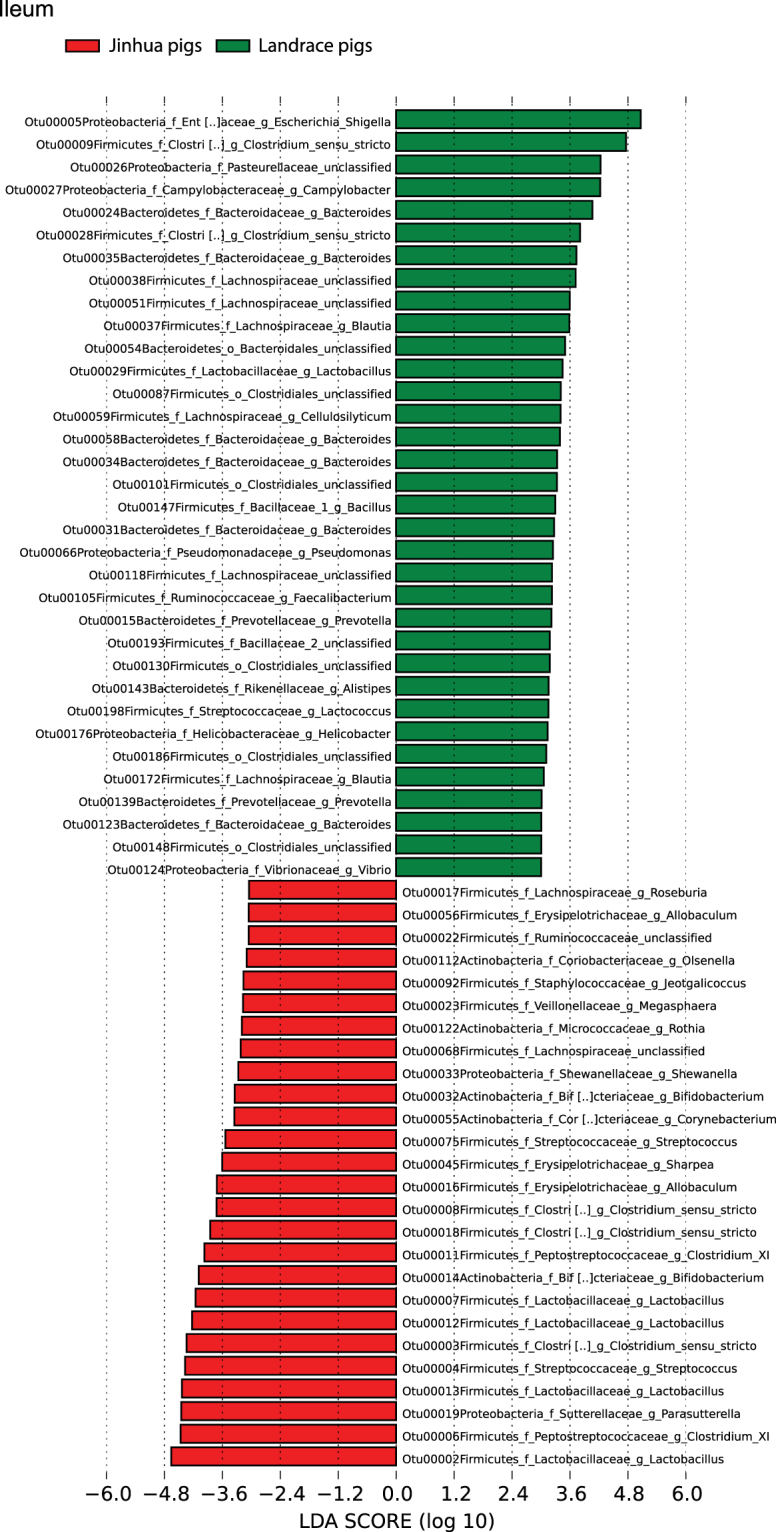
Bacterial taxa differentially represented in ilum in Jinhua and Landrace pigs identified by LEFSe using a LDA score threshold of >2.0.

To identify enrichment of specific bacterial taxa between the two breeds, we performed LEfSe^24^, which not only emphasizes statistical significance, but also biological consistency. Based on the logarithmic LDA score of 2.0 as the cutoff, a number of OTUs were differentially represented between Jinhua and Landrace pigs (Fig. 7). While many were uniquely enriched in a specific intestinal segment of a breed, a dozen OTUs were more prevalent in more than one segment. For example, a member of the *Escherichia* species (OTU #5), which accounted for 23–28% of all bacteria (Fig. 6), was over-represented in both the jejunum and ileum of Landrace pigs (Fig. 7, Figure S3). A member of the *Prevotella* genus (OTU #15) was also dominant in both the cecum and colon of Landrace pigs. On the other hand, a *Bifidobacterium* species (OTU #14) was more prevalent in the duodenum, ileum, and cecum of Jinhua pigs, and a *Lactobacillus* species (OTU #13) was dominant throughout the small intestinal tract of Jinhua pigs. Two members in the genus of *Clostridium sensu stricto* (cluster I; OTU #3 and #18) were more abundantly associated with Jinhua pigs in the ileum and jejunum, while OTU #18 was more dominant in the cecum and colon of Jinhua pigs that those of Landrace pigs. Similarly, an *Allobaculum* species (OTU #16) showed over-representation in the duodenum, ileum, and cecum of Jinhua pigs.

Interestingly, a few OTUs showed a different pattern of dominance in the two breeds (Fig. 7, Figure S3). For example, while a member of *Lactobacillus* (OTU #2) was more abundant in both the duodenum and colon of Landrace pigs, it became more prevalent in the ileum of Jinhua pigs. Another *Lactobacillus* (OTU #7) was clearly abundant in the duodenum of Landrace pigs, but also had a prevalence in the ileum of Jinhua pigs. A *Megasphaera* species (OTU#16) was over-represented in the cecum of Landrace pigs, but was more dominant in the duodenum of Jinhua pigs.

### Differences in the functional potential of intestinal bacterial community in Jinhua and Landrace pigs

**Figure 8:**
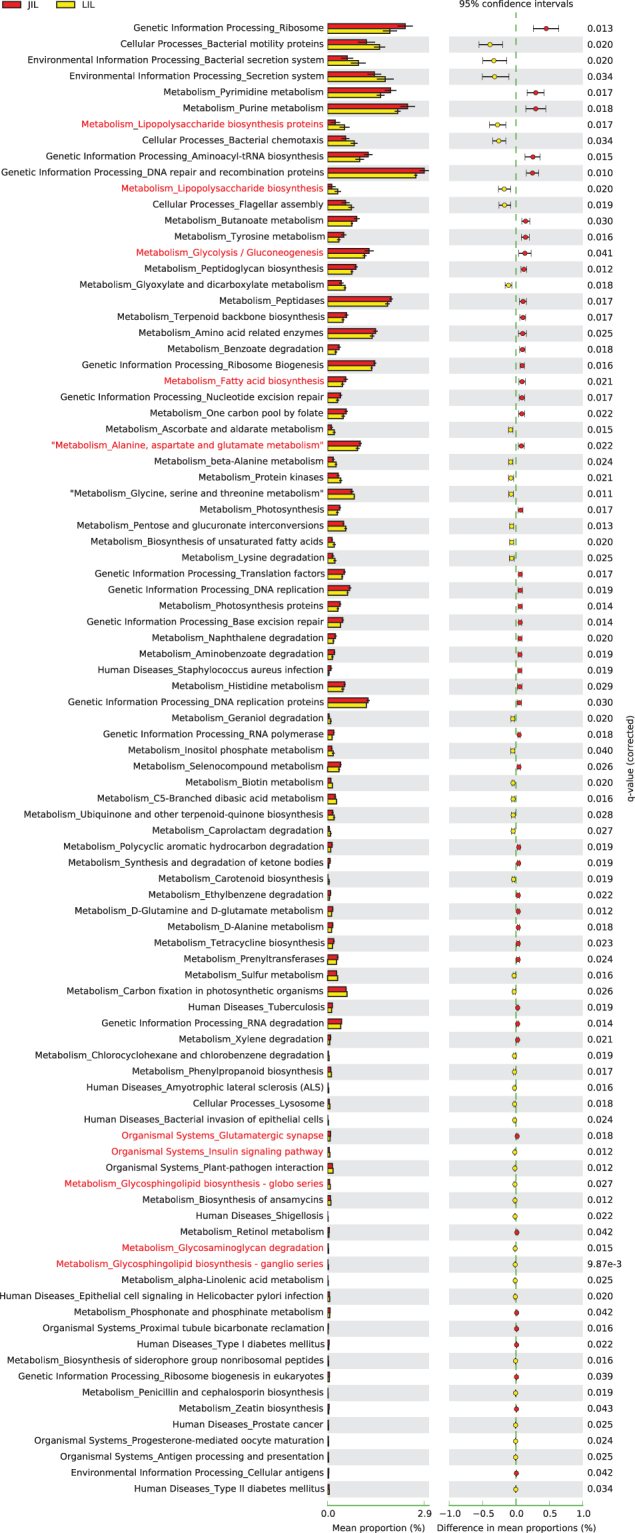
Predicted function of ileal microbiota between Jinhua (red bar) and Landrace (yellow bar) pigs. The third level of KEGG pathways were shown in the post-hoc plot. The significance of the gene distribution between the groups were performed using ANOVA test with a *P* value < 0.05.

To further predict how enriched bacteria potentially contribute to the differences in the host phenotype of the two breeds, i.e. growth and adipogenesis specifically, we performed PICRUSt analysis of the microbiome in different segments of the intestinal tract^25^. Many bacterial genes that could potentially trigger inflammatory responses (such as LPS biosynthesis, motility, secretion, chemotaxis, and flagellar assembly) were predicted to be enriched in both the ileum and jejunum of Landrace pigs. On the other hand, the bacterial genes involved in the metabolism of fatty acids, proteins, amino acids, nucleotides, and carbohydrates were predicted to be enriched in Jinghua pigs (Fig. 8, Figures S4 and S5).

## Discussion

Recent studies in the swine gastrointestinal microbiome have greatly expanded our knowledge on the role of the gut microbiome in different phenotypes. However, most of these studies focused on fecal microbiome, with very few studies involving the microbiome comparison between different sections of the intestinal tract. Particularly, the composition and function of duodenal and jejunal microbiome of swine remains elusive. Although a recent study attempted to define the core microbiome in different sections of the gastrointestinal tract by a meta-analysis of 20 publications, a huge variation among different studies was revealed^27^. In this study, we characterized the gut microbiota structure and predicted function in five different segments of the intestinal tract including the duodenum, jejunum, ileum, cecum, and colon in both Jinhua and Landrace pigs. In doing so, we provided a comprehensive view of the biogeography of the swine gut microbiome.

We revealed that the large intestinal microbiome was different from the small intestinal microbiome in both Jinhua and Landrace pigs. This is especially the case when comparing the cecal microbiome with other small intestinal sections. This is not surprising when considering the different physiologies, functions and ecological environments of the different intestinal sections. To date, very limited information is available on duodenal microbiome. In this study we found that the microbiome in the duodenum is distinct from that in the jejunum of Landrace pigs; however, such a difference was not noticeable within Jinhua pigs. A meta-analysis indicated that *Lactobacillus* is enriched in the stomach, possibly due to their acid tolerance in the low pH environment^27^. Because of the presence of *Lactobacillus* in all types of GI-tract samples, it was postulated that the stomach might serve as a source of these bacteria in other gut sections^27^. Notably, in this study, *Lactobacillus* was more abundant in the duodenum than in any other sections of both breeds, suggesting that the higher abundance of *Lactobacillus* in the duodenum is likely to originate from the stomach.

We showed in this study that the large intestine (e.g., cecum and colon) harbors more diverse microbiome than the small intestine (e.g., duodenum, jejunum, and ileum) in both Jinhua and Landrace pigs, which is consistent with previous studies^27,28^. However, significant differences in microbial diversity exist between the two breeds within specific intestinal segments. For example, Jinhua pigs harbor more diverse microbial community in the duodenum, jejunum and cecum than Landrace pigs. Since the Jinhua pig is an obese breed, our data appears to be contradictory to earlier observations that obese people have less diverse fecal bacterial community than lean people^29^. However, this apparent discrepancy may be due to the fact that pigs are different from humans and the fact that fecal microbiome does not necessarily reflect intestinal microbiome. In fact, relative to Landrace pigs, Jinhua pigs had a slightly reduced diversity in colonic microbiota, which agrees with previous reports describing colon microbiota as similar to fecal microbiota^30^. The more diverse microbiome in the small intestine and cecum of Jinhua pigs might help with nutrient digestion, energy harvest, and fat deposition in this obese pig breed. Consistently, analysis of the metabolic potential of the ileal microbiome by PICRUSt revealed an enrichment in fatty acid biosynthesis pathways in the obese Jinhua breed. Since fatty acid synthesis has been linked to obesity and type 2 diabetes in human adipose tissue^31^, our data suggests that the ileal microbiome may contribute to excess energy intake and increased body fat mass in the obese Jinhua pigs. Our study provides clear evidence that the microbiome in other intestinal sections is worth more attention in obesity studies.

OTUs associated with *Escherichia* were abundant in the small intestinal sections (e.g., jejunum and ileum) in the Landrace pigs which is consistent with previous reports^28,30^. However, these OTUs were significantly less abundant in the small intestine of Jinhua pigs, which partially explains the higher abundance of genes in Landrace pigs that potentially trigger inflammatory responses such as LPS biosynthesis, motility, secretion, chemotaxis, and flagellar assembly predicted by PICRUSt. Chronic inflammation has been associated with obesity. In a recent study, Yang *et al*. found that *Escherichia spp*. was significantly enriched in Laiwu pigs, another Chinese indigenous breed with extremely high intramuscular fat content^28^. The discrepancy in the abundance of *Escherichia* between the two obese breeds, Jinhua (this study) and Laiwu (Yang *et al*.), might be due to the differences in genetics, diet, environment, and developmental stages. Of note, there are several limitations of this study. First, despite PICRUSt being used in a wide range of host-microbiota studies, it was mainly developed by using reference genomes tailored to humans. Second, there might be several factors affecting the differences in the biogeography of the gut microbiota between the Jinhua and Landrace piglets such as the sow’s microbiota, which might contribute to the differences in the piglets’ gut microbiota by serving as an initial inoculator of the piglets’ gut microbiota. Furthermore, significantly differences in body weight observed between the Jinhua and Landrace piglets, very likely due to the differences in breeds, might also affect the gut microbiota. Nevertheless, the purpose of this study is not to pinpoint the origins and confounders of the swine gut microbiota, rather, we want to describe the longitudinal variation along the GI-tract microbiota and the cross-sectional variation between the two breeds. Further experiments are desired to determine if the intra- and inter-breed variation in the biogeography of the intestinal microbiome explains the differences in growth performance and fat deposition between different swine breeds.

## Electronic supplementary material

Supplemental information
