# Supplementary material for: Comparative biogeography of the gut microbiome between Jinhua and Landrace pigs
Source: Sci Rep. 2018 Apr 13;8:5985. doi: 10.1038/s41598-018-24289-z (PMC5899086; doi:10.1038/s41598-018-24289-z)
Supplement: Supplementary file 1 — Supplemental information [file 41598_2018_24289_MOESM1_ESM.docx]

**Comparative biogeography of the gut microbiome between Jinhua and Landrace pigs**

Yingping Xiao^1^, Fanli Kong^2^, Yun Xiang^3^, Weidong Zhou^4^, Junjun Wang^5^, Hua Yang^1^*, Guolong Zhang^6^*, Jiangchao Zhao^2^*.

Supplemental tables and figures

Table S1. ANOSIM analysis of dissimilarities in gut microbiota between breeds

Figure S1


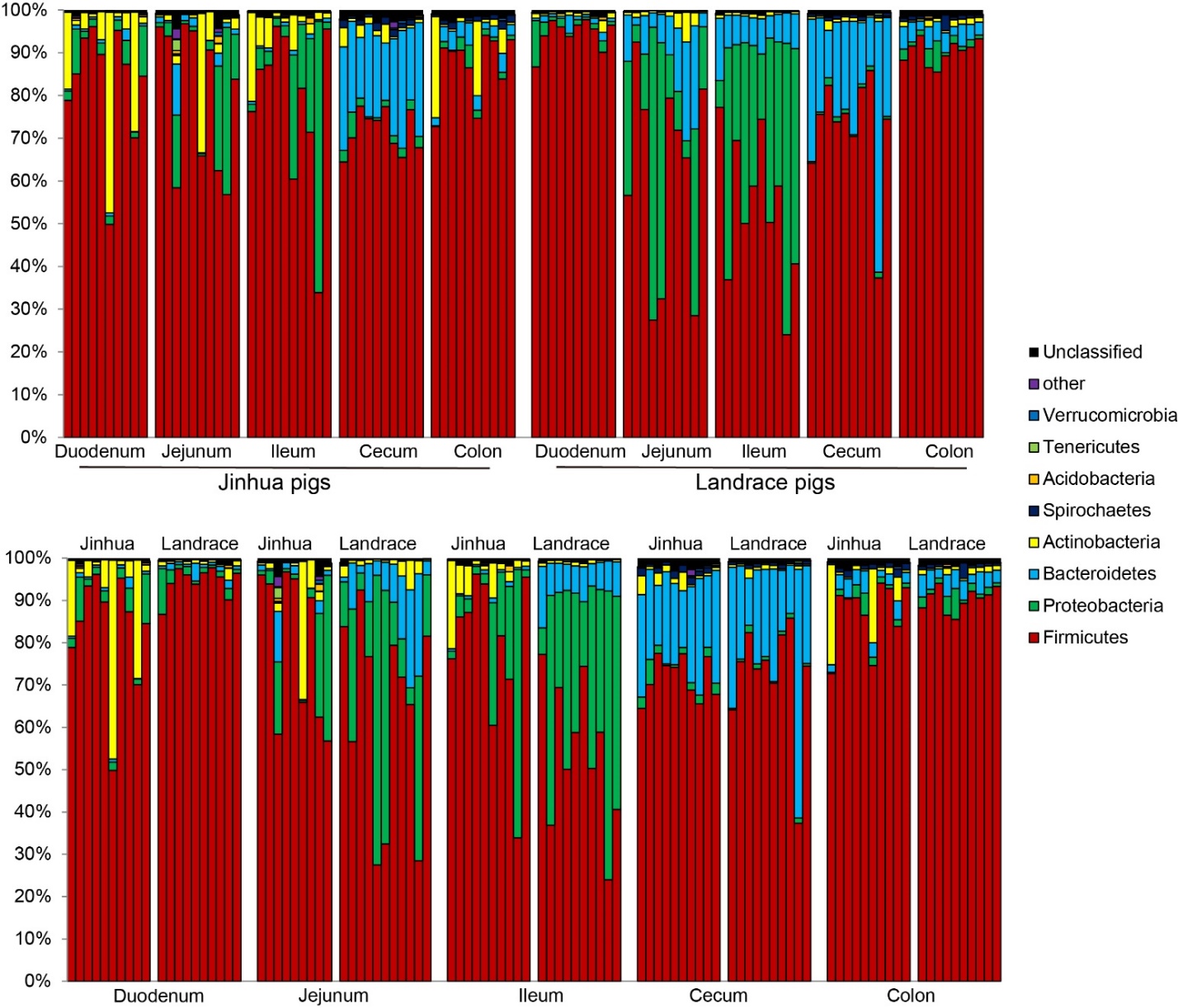


Figure S1. Community composition at the phylum level. Each bar represents the relative abundance of a phylum.

Figure S2


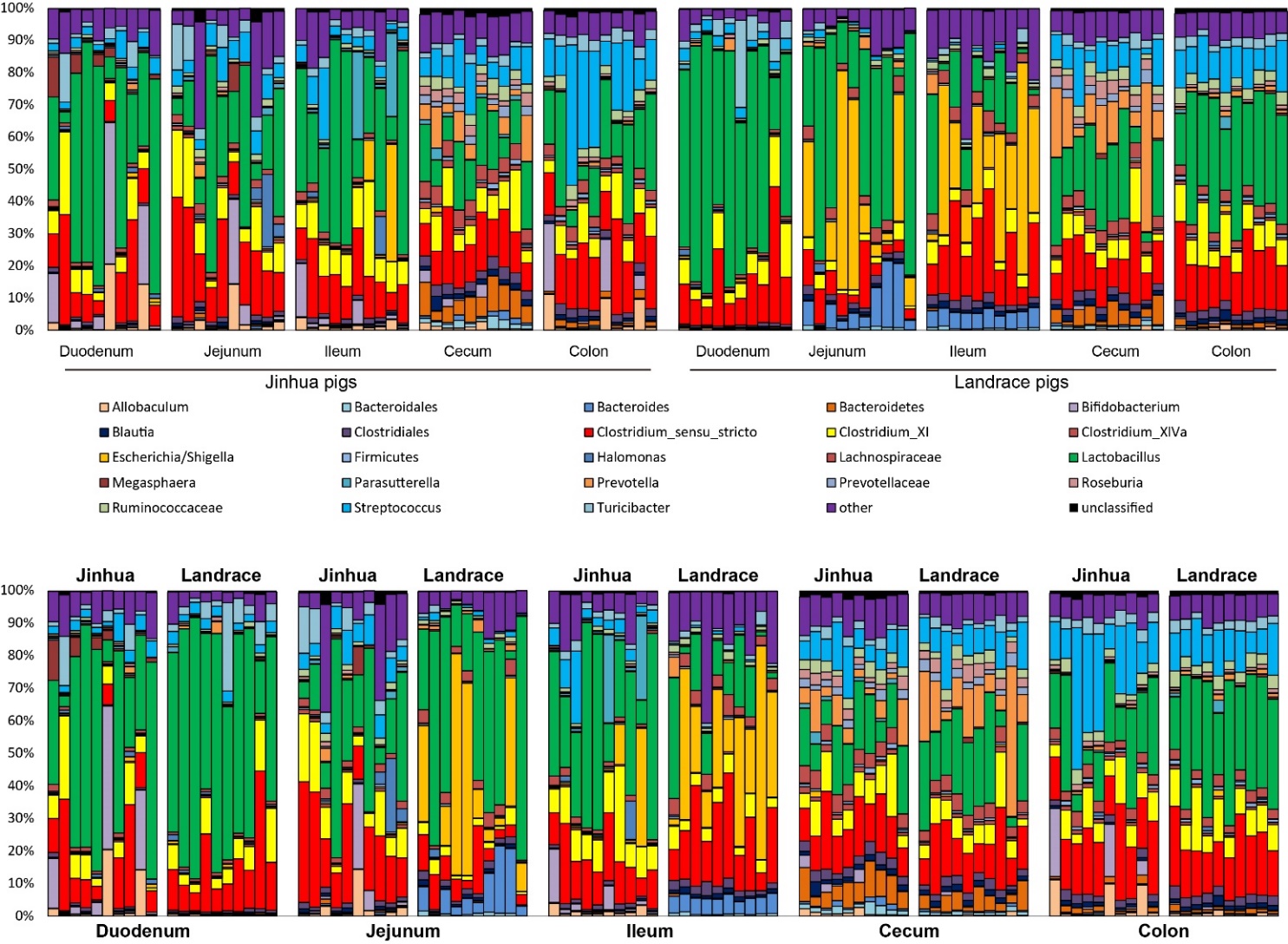


Figure S2. Community composition at the genus level. Each bar represents the relative abundance of a genus.

| 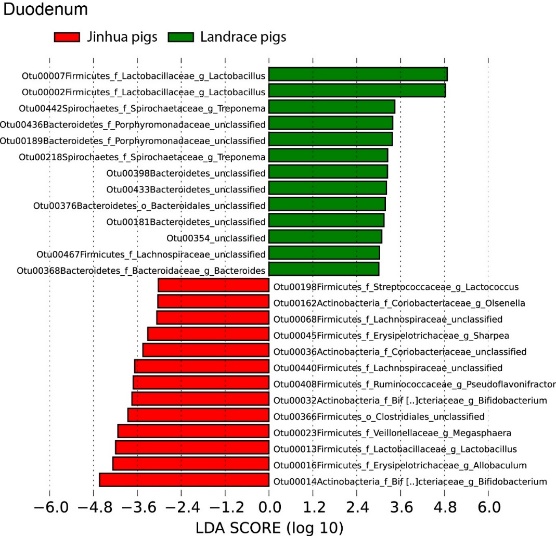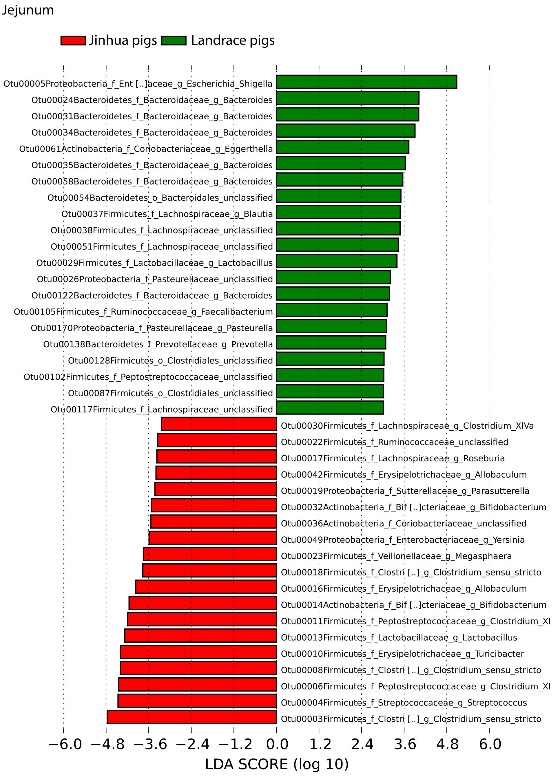 | 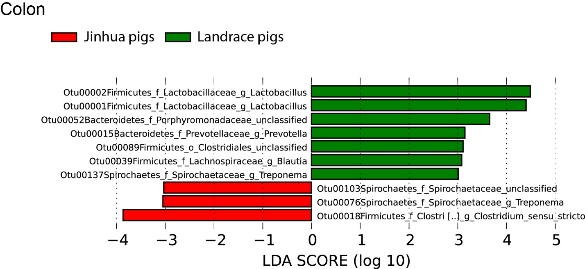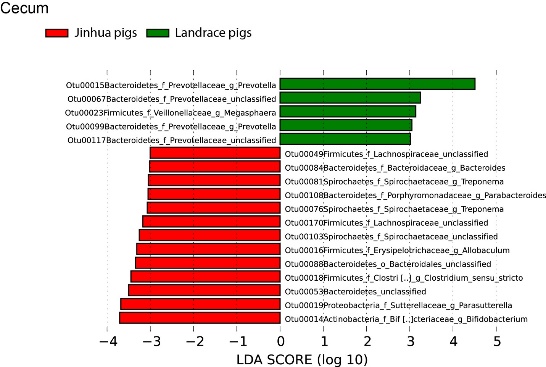 |
| --- | --- |
|  |  |
|  |  |
|  |  |
|  |  |

Figure S3. Bacterial taxa differentially represented in different intestinal segments between Jinhua and Landrace pigs identified by LEFSe using a LDA score threshold of >2.0.

Figure S4.


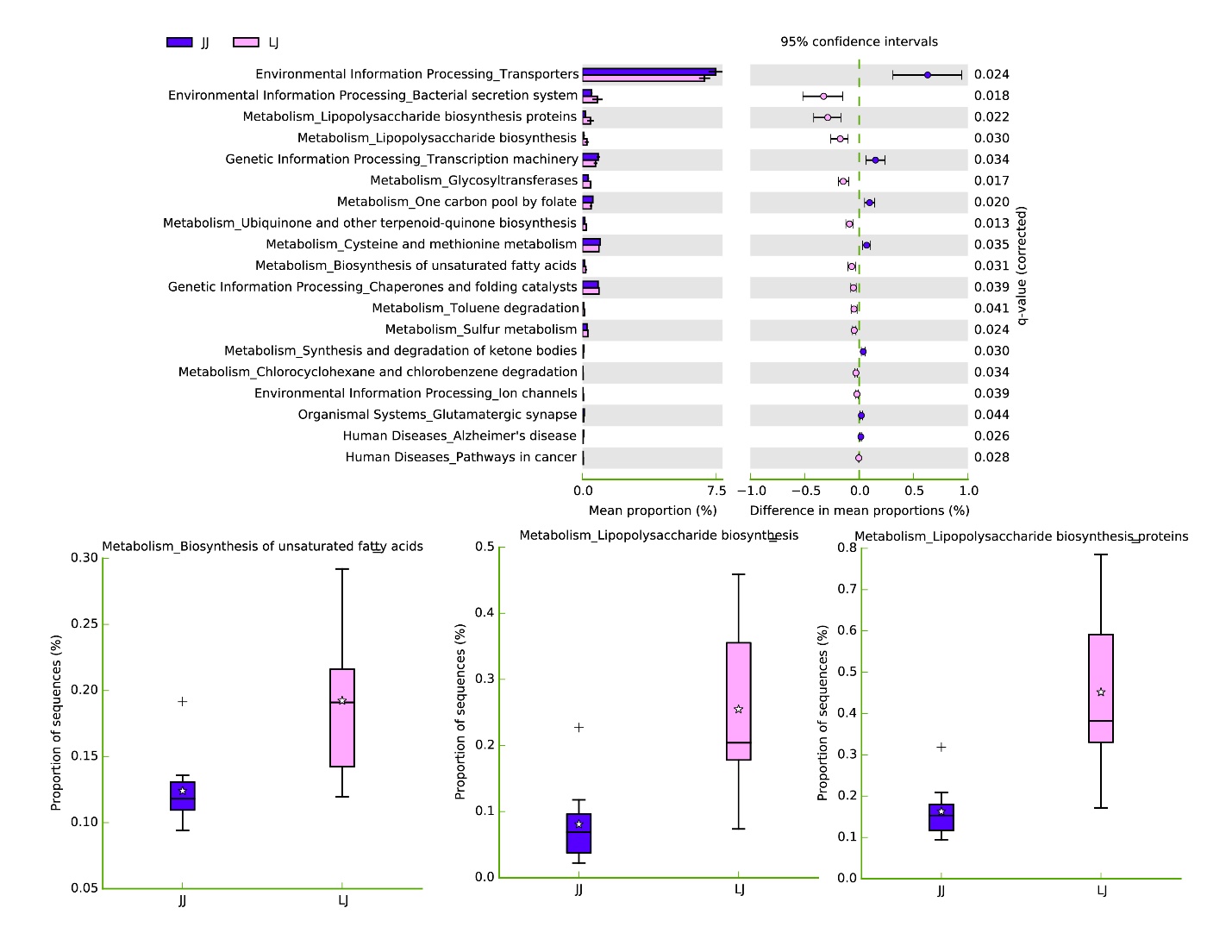


Figure S4 Predicted function of jejunal microbiota between Jinhua and Landrace pigs. The third level of KEGG pathways were shown in the post-hoc plot. The significant test of the gene distribution between groups were performed using ANOVA test with a p value <0.05.


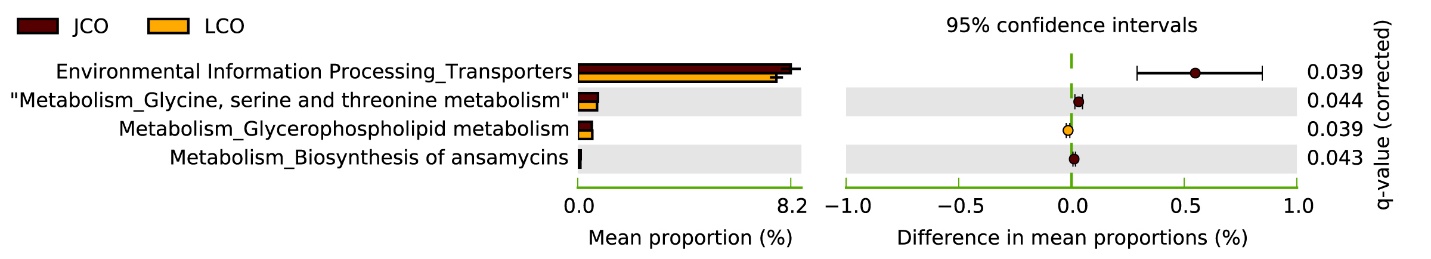


Figure S5 Predicted function of conlonic microbiota between Jinhua and Landrace pigs. The third level of KEGG pathways were shown in the post-hoc plot. The significant test of the gene distribution between groups were performed using ANOVA test with a p value <0.05.
